# Parkinson's disease prevalence in Fabry disease: A survey study

**DOI:** 10.1016/j.ymgmr.2017.10.013

**Published:** 2017-11-09

**Authors:** Adina H. Wise, Amy Yang, Hetanshi Naik, Chanan Stauffer, Natasha Zeid, Christopher Liong, Manisha Balwani, Robert J. Desnick, Roy N. Alcalay

**Affiliations:** a Department of Neurology, Columbia University Medical Center, 710 W. 168th St., New York, NY 10032, United States; b Department of Genetics and Genomic Sciences, Icahn School of Medicine at Mount Sinai Hospital, 1428 Madison Ave, Atran Building, 1st Floor, New York, NY 10029, United States

**Keywords:** ISMMS, Icahn School of Medicine at Mount Sinai, Parkinson's disease, Fabry disease, Lysosomal storage disorder, Alpha-galactosidase A, GLA

## Abstract

Recent research has suggested a possible link between Parkinson's disease (PD) and Fabry disease. To test this relationship, we administered a self-report and family history questionnaire to determine the prevalence of PD in Fabry disease patients and family members with likely pathogenic alpha-galactosidase A (GLA) mutations. A total of 90 Fabry patients (77 from the online survey and 13 from the Icahn School of Medicine at Mount Sinai (ISMMS)) were included in the analysis. Two of the Fabry disease patients who completed the online survey were diagnosed with PD (2/90, 2.2%). Among probands older than 60, 8.3% (2/24) were diagnosed with PD. Using Kaplan Meier survival analysis, the age-specific risk of PD by age 70 was 11.1%. Family history was available on 72 Fabry families from the online study and 9 Fabry families from ISMMS. Among these 81 families, 6 (7.4%) had one first degree relative who fit the criteria for a conservative diagnosis of PD. The results of this study suggest that there may be an increased risk of developing PD in individuals with GLA mutations, but these findings should be interpreted with caution given the limitations of the study design.

## 1 Introduction

The link between Parkinson's disease (PD), and the glucocerebrosidase (GBA) mutations causing Gaucher disease highlights the potential role of lysosomal dysfunction in the pathogenesis of PD [1]. Gaucher's disease is caused by diminished glucocerebrosidase enzymatic activity, resulting from loss of function mutations in the GBA gene. While GBA mutations are the most common genetic alterations in PD, other lysosomal enzymes have also been associated with PD, including the gene causing Niemann-Pick A and B (SMPD1) [2], [3], [4] and SCARB2 [5], which encodes the glucocerebrosidase chaperone LIMP-2.

Recent research has also suggested a link between PD and Fabry disease (FD), an X-linked lysosomal storage disorder caused by the deficient activity of alpha-galactosidase A (α-Gal A). Wu et al. (2011) [6] examined α-Gal A activity in white blood cells, and found that PD patients had lower α-Gal A activity compared to controls. In addition, Nelson et al. (2014) [7] examined brains of a Fabry mouse model, and found aggregates of alpha-synuclein, the protein accumulation most often associated with PD in human brains, in the white matter pons. Two case reports of Parkinsonism in Fabry disease patients have been published. Orimo et al. (1994) [8] describes a 68-year old man with a 5-year history of Parkinsonism. Upon autopsy, Fabry disease was confirmed, but brain autopsy was not performed. Buechner et al. (2006) [9] described a 57-year old woman with progressive bradykinesia and rigidity consistent with Parkinsonism, who was levodopa responsive. She had clinical findings consistent with the FD classic phenotype, including acroparesthesias, corneal opacities and mitral valve prolapse, and eventually received a diagnosis of Fabry disease after a brain MRI showing leukoencephalopathy with multifocal ischemic lesions led to the discovery of low α-Gal A activity and the R301P mutation in the alpha-galactosidase A (GLA) gene.

Given these findings, we surveyed Fabry patients with a self-report and family history questionnaire to determine the prevalence of PD in Fabry disease patients and family members with GLA mutations.

## 2 Material and methods

Two methodologies were used to recruit subjects: 1) An online, anonymous version of a validated family history of Parkinson disease questionnaire [10] was created using Qualtrics software. This survey was distributed to members of the Fabry disease community, via the National Fabry Disease Foundation and the Fabry Support and Information Group. The link was delivered to members of these groups through e-newsletters and mailings, which were sent directly from the group leaders in order to maintain subject anonymity. The researchers had no direct contact with study participants and no identifying information was collected through the questionnaire. This questionnaire was determined to be exempt from human subjects regulation by the Columbia University Institutional Review Board (IRB). 2) Patients who are seen at the Lysosomal Storage Disease Program at the Icahn School of Medicine at Mount Sinai (ISMMS) were invited to participate in the study and complete the same family history questionnaire as described above, either in person during their clinic visits, or by phone or mail. All Fabry disease patients at Mount Sinai were mailed a recruitment letter with information about the study and were informed that a member of the study team may reach out to them. These individuals were also given the option of refusing further contact with this study. Recruitment of these participants was not anonymous. This study was approved by the ISMMS IRB.

To ascertain Fabry disease status, participants were asked whether they identify as a Fabry disease patient, a first-degree family member of a Fabry disease patient, or both, and were asked to provide their specific GLA mutation, if known. The survey then asked about participant's personal and family histories of PD, specifically if and when the individual had been diagnosed with PD. Participants were also given an option of adding free text information.

To ascertain family history of PD, we used a previously validated family history questionnaire [11]. For each first-degree relative, the survey asked if the relative had a Fabry disease mutation and if he/she had been diagnosed with PD. If the individual had been diagnosed with PD, the survey asked a series of follow-up questions including: 1) if this diagnosis was made by a neurologist, 2) if the individual was treated with carbidopa-levodopa, 3) the age at diagnosis of PD. Regardless of whether a PD diagnosis was reported, participants were asked to report if any first-degree relatives exhibited common symptoms of PD (resting tremor, shuffling gait, stooped posture decreased arm swing, rigidity), and, if so, which was the initial symptom. Similar questionnaires were used to estimate PD penetrance in other genes linked to PD, including LRRK2 and GBA [12], [13]. We follow a previously validated “conservative” algorithm of interpretation of the same family history questionnaire published by Marder et al. [11], which had 95.5% sensitivity and 96.2% specificity for PD for the proband's family history report. Age and gender were also collected for all participants and first-degree family members. A copy of the questionnaire, which was used for the online survey is attached (supplementary document).

### 2.1 Statistical analysis

Cohort characteristics and demographic information are reported using means and standard deviation (SD) for continuous variables and percent for categorical variables. We attempted to address the possibility that patients filled out the online survey more than once by excluding entries which appeared to be duplicates, based on overlapping age, year of birth, sex, family members reported to have Fabry disease and/or reported Fabry mutation. The incidence of PD among participants with *GLA* mutations aged 60 or older was also calculated. Kaplan-Meier survival curves were used to estimate age-specific Parkinson disease penetrance among subjects with Fabry disease mutations.

## 3 Results

We received a total of 87 responses to the online survey between October 2016 and July 2017. Three respondents were excluded as they could not have inherited the Fabry mutation based on X-linked inheritance pattern and three additional respondents were excluded due to inability to confirm mutation status (female respondents without signs of Fabry disease who report a mother or brother with Fabry). The remaining 81 respondents all reported Fabry disease mutations; 77 were self-reported Fabry disease patients and 4 were obligate female heterozygotes who did not identify as having Fabry symptoms (2 females with sons who have been diagnosed with Fabry disease, 1 female with a son and mother who have been diagnosed with Fabry disease and 1 female with a father diagnosed with Fabry disease).

We conducted the analysis excluding an additional 4 entries (one with the p.E79X mutation, two with the p.Y134X mutation and one with the p.E59V variant), which are likely to be duplicates based on genotype and age, including one participant with the p.E59V variant who reported PD. Of the 77 remaining respondents with Fabry disease mutations, 52 were female (67.5%) and 25 were male (32.5%); the mean age was 52.1. Precise genotype was provided by 38 respondents (51.9%). Of those that reported mutation status, in addition to those excluded, two listed each of the following mutations: p.R227X, p.D264Y, p.I270T, p.L32P, and p.W287C.

An additional 13 individuals were recruited to the study through the ISMMS, 11 female (84.6%) and 2 male (15.4%); the mean age of this cohort was 35.2. All of these participants have molecular confirmation of Fabry disease. None of these individuals reported a history of PD in themselves or in their family members. Genotype information was available for all of these participants, and four have presumed *de novo GLA* mutations. All parents of these four patients were tested negative for the mutations. Based on age and genotype, none of the individuals recruited from ISMMS were duplicates with the online questionnaire.

A total of 90 Fabry individuals with Fabry mutations (77 from the online survey and 13 from ISMMS) were included in the analysis. Two of the individuals with Fabry disease mutations were diagnosed with PD (2/90, 2.2%). Among probands older than 60, 8.3% (2/24) were diagnosed with PD. Using Kaplan Meier survival analysis, the age-specific risk of PD by age 70 was 11.1%. One FD/PD respondent reported the p.Y134X mutation and one reported the p.E59V variant. Of note, one additional entry reported a diagnosis of PD and the p.E59V mutation, however, due to a high suspicion of this being a duplicate entry, as described above, we chose to be conservative in our calculations and therefore did not count this as a unique case of PD. In the literature, Y134X has previously been associated with the classic Fabry phenotype [14], [15]. The p.E59V variant is not registered in publically-available mutation databases, but a missense mutation at the same position, E59K, causes classic Fabry disease [14]. In addition, *in silico* prediction models such as MutationTaster and Provean predict this variant to be “disease causing” and “damaging” [16].

Family history was available for 72 unique Fabry families from the online study and 9 Fabry families from ISMMS (two respondents from the online survey cohort were excluded due to adoption status and 3 respondents from the online survey were likely from the same family based on genotype and family tree. Four participants were excluded from the ISMMS cohort due to known *de novo* mutation status). Among these 81 families, 4 (4.9%) had one first-degree family member who was diagnosed with PD by a clinician and 6 (7.4%) had one family member who was diagnosed with PD based on the conservative algorithm of Marder et al. [11]. One of these respondents was a 50-year-old female Fabry patient who is currently being evaluated for a “movement disorder.” As follow-up exams were not possible, these individuals could not be counted as PD patients, regardless of the number of symptoms reported.

## 4 Discussion

In this report, we aimed to estimate the age-specific frequency of PD in Fabry patients. Among responders, 2 patients were diagnosed with PD (risk of 11.1% by age 70) and 6 families (out of 81, 7.4%) had one first degree relative who fit the conservative criteria for a diagnosis of PD. While estimates of lifetime or age-specific PD risk vary widely, previous epidemiological studies have estimated PD risk at 1.0–1.5% by age 65 [17]. The results of this study suggest that there may be an increased risk of developing PD in individuals with *GLA* mutations, but these findings should be interpreted with caution given the limitations of the study design. Collecting anonymous data online allowed for a greater response rate. However, respondents could not be contacted to clarify responses or to collect additional follow up information. Also, the participants could not be examined by a movement disorders specialist. Though medical records of GLA mutation carriers were only available for the 13 individuals recruited directly from Mount Sinai, we may be reasonably confident that the respondents who self-reported as Fabry disease patients or first-degree family members of Fabry disease patients have identified themselves accurately, as the survey was distributed only through Fabry disease support groups and was not made available to the general public.

A major limitation of the online, anonymous recruitment methodology was the possibility of respondents completing the survey more than once. We chose a conservative approach of removing suspected duplications, including a case who reported PD, as there was no way to return to the participant to confirm or refute duplication. This methodology also made it difficult to know which participants, if any, represented members of the same Fabry family. Furthermore, this study design did not include a clinical exam to confirm diagnosis of PD, or lack thereof. It is possible, therefore, that we underestimate the association between FD and PD. An additional limitation of this study is the response rate. It is impossible to conclude the exact response rate, but for comparison, a recent study which utilized an identical online, anonymous recruitment methodology, but asked about women's health issues in Fabry disease, collected approximately 100 responses, whereas our study had 87. A 2006 study investigating the experience of pain in Fabry disease patients also utilized an anonymous questionnaire sent to Fabry support group members and received 97 responses [18].

None of the Fabry patients who were recruited directly through ISMMS reported symptoms of PD in family members; however, this may be due to the fact that this cohort was significantly younger than the online cohort (35.2 *vs.* 52.1, *p*-value < 0.001). Another cohort of Fabry patients was previously reported. Lohle et al. (2015) [19] examined 110 individuals with *GLA* mutations (60 heterozygous females and 50 affected males) and found that, while individuals with confirmed *GLA* mutations do present some elements of a bradykinetic motor phenotype, including slower gait and lower hand speed, these individuals did not report the classic prodromal features of PD (hyposmia/anosmia, autonomic dysfunction, REM Sleep Behavior Disorder), as previously found in individuals with *GBA* mutations [20].

The estimated median cumulative survival for Fabry disease patients, based on questionnaire data prior to enzyme replacement therapy [21], is 50 years for affected males and 70 years for heterozygotes [22], [23]. As PD is a disease of older adults, with a peak incidence of onset between 70 and 79 years, many Fabry patients may not live long enough to develop PD [24]. Considering that life expectancy in Fabry disease has improved with enzyme replacement therapy, it is possible that treatment and prolonged life expectancy could uncover a potential association with PD. Alternatively, transient cerebral ischemia and strokes are common in Fabry patients, secondary to atrial fibrillation [25], and the PD symptoms experienced by individuals with Fabry could be secondary to cerebral infarctions, leading to the development of vascular Parkinsonism.

## 5 Conclusion

The results of this study highlight the necessity for further research to elucidate the relationship between Fabry disease and Parkinson's disease. The intention of this study was to establish whether more rigorous investigation of the impact of *GLA* mutations on the development of PD is indicated. Based on the implications of this initial signal, future studies should focus on larger cohorts, in-person examination of individuals with Fabry disease by a movement disorders neurologist, and related biomarker studies including alpha-galactosidase A activity and *GLA* mutation analysis. Finally, neuropathological studies of Fabry disease patients, including those with Parkinsonism, are required to evaluate whether Parkinsonism in Fabry disease is associated with alpha synuclein deposition in Lewy bodies, similar to idiopathic PD and Gaucher-related PD.

## Appendix A Supplementary data

This questionnaire was provided to all study participants. Image 1
