# Supplementary material for: Parkinson's disease prevalence in Fabry disease: A survey study
Source: Mol Genet Metab Rep. 2017 Nov 9;14:27–30. doi: 10.1016/j.ymgmr.2017.10.013 (PMC5683804; doi:10.1016/j.ymgmr.2017.10.013)
Supplement: Supplementary file 1 — This questionnaire was provided to all study participants. [file mmc1.pdf]

Introduction

The purpose of this research study is to describe the prevalence of Parkinson disease in patients with Fabry disease and their first-degree family members (parents, children, siblings). You are being asked to take part in this research study because you have been diagnosed with Fabry disease or because you have a first-degree family member who has been diagnosed with Fabry disease.

Being in a research study is completely voluntary. You can choose not to be in this research study. You can also say yes now, and change your mind later.

If you agree to take part in this research, you will be asked to complete a questionnaire with questions about general health information and your family history of Parkinson disease. Your participation in this study will take about 15 minutes.

You can choose not to answer any question you do not wish to answer. You can also choose to stop taking the survey at any time.

You must be a Fabry disease patient or first-degree family member of a Fabry disease patient to participate. If you do not have Fabry disease, or do not have a first-degree family member with Fabry disease, please stop now.

The possible risks to you in taking part in this research are:
- You may feel uncomfortable answering some of the questions
- Someone else might find out you were in a research study

The possible benefits to you for taking part in this research are:
- Increase the medical understanding of individuals with Fabry Disease with respect to the development of Parkinson disease.

To protect your identity as a research subject, no identifiable information will be collected and in any publication about this research, no private information will be used.

If you have any questions about this research, please contact the Principal Investigator, Dr. Amy Yang at 212-241-0915 or amy.yang@mssm.edu. You can also call the Program for the Protection of Human Subjects Office at 212-824- 8200 if you have any concerns about this research.

Demographic Information

Demographic Information:

What is your age?

[ ]

What is your year of birth?

[ ]

What is your sex?

- ○ Male
- ○ Female

Are you adopted?

- ○ Yes
- ○ No

Fabry Disease Information:

Fabry Disease Information:

Please select the option(s) that best describes you:

- ☐ I have been diagnosed with Fabry disease
- ☐ I am a first-degree relative (parent, sibling or child) of someone who has Fabry disease

Please select the family member(s) diagnosed with Fabry disease (please select all that apply).

- ☐ Mother
- ☐ Father
- ☐ Son
- ☐ Daughter
- ☐ Brother
- ☐ Sister
- ☐ [ ] Other

Please provide your Fabry disease genotype, if known:
(ex: G43R, g11185-delT)

[ ]

PD Personal

Personal History of Parkinson Disease:

Have you been diagnosed with Parkinson disease?

- ○ Yes
- ○ No

What year were you diagnosed with Parkinson disease?

[ ]

Have any of your family members been diagnosed with Parkinson's disease?

- ○ Yes
- ○ No

- ○ Maybe (please explain) ______

Please select all of the family members who have been diagnosed with Parkinson's disease.

- ☐ Mother
- ☐ Father
- ☐ ______ Sibling(s) (please enter number of siblings with Parkinson's disease)
- ☐ Maternal Grandmother
- ☐ Maternal Grandfather
- ☐ Paternal Grandmother
- ☐ Paternal Grandfather
- ☐ Other (please describe) ______

Block 8

The remainder of this survey will ask you to answer the same series of questions related to the symptoms and diagnosis of Parkinson disease for each of your parents, children, and siblings. Please select the individual about whom you are answering this series of questions, and then proceed.

If the family member is deceased, please answer the questions based on your memory of the person, to the best of your ability (please note that all questions are written in the present-tense).

PD History: Parents

The following information pertains to my:

- ○ Mother
- ○ Father

Year of Birth

Does or did this person have the mutation in the Fabry gene or have symptoms of Fabry?

- Yes
- No
- I don't know

Is this person living?

- Yes
- No

Please indicate age at death:

What was the cause of death?

- Unknown
- Heart disease
- Cancer
- Cerebrovascular disease (stroke)
- Chronic lower respiratory disease
- Accident (unintentional injury)
- Diabetes
- Alzheimer disease
- Influenza and pneumonia
- Kidney disease
- Septiciemia
- Intentional self-harm (suicide)
- Chronic liver disease and cirrhosis
- Essential hypertension and hypertensive renal disease
- Parkinson disease
- Homicide
- Natural causes
- Other

Was an autopsy performed?

- Yes
- No
- Unsure

Did the autopsy indicate Parkinson's disease?

- Yes
- No
- Unsure

Has he/she been diagnosed with Parkinson's disease by a physician?

- Yes
- No
- ____________ Unsure

If yes, was the physician a neurologist?

- Yes
- No
- Unsure

How old was he/she when the diagnosis was made?

____

Does he/she take Sinemet (Levodopa/Carbidopa)?

- Yes
- No
- ____________ Unsure

Does he/she exhibit any of the following symptoms? Please check all that apply.

- ☐ Tremor at rest or when sitting quiety
- ☐ Shuffling gait and/or steps that are short and tiny
- ☐ Stooped posture
- ☐ Arms that do not swing when he/she walks
- ☐ Muscular rigidity or slow/stiff movements

If he/she has any of the above symptoms, at what age did they begin?

____

If this person has any of the symptoms listed below, which were the first to develop? (Please select all that apply).

- ☐ rest tremor
- ☐ action tremor
- ☐ slowness
- ☐ stiffness
- ☐ balance problem
- ☐ shuffling gait
- ☐ decreased arm swing
- ☐ decreased facial expression
- ☐ decreased volume of speech
- ☐ dystonia
- ☐ stooped posture
- ☐ small handwriting
- ☐ freezing
- ☐ memory loss
- ☐ numbness
- ☐ hallucinations
- ☐ depression
- ☐ leg dragging
- ☐ other __________

Thank you. You will now be asked the same series of questions about your other parent. If you have already completed these questions for both parents, you will be asked to provide information about your children and siblings.

Siblings

How many siblings do you have?

[ ]

PD History: Siblings

Please answer the following questions for each of your siblings.

Is this person male or female?

- ○ Male
- ○ Female

Year of Birth

[ ]

Does or did this person have the mutation in the Fabry gene or have symptoms of Fabry?

- ○ Yes
- ○ No
- ○ I don't know

Is this person living?

- ○ Yes
- ○ No

Please indicate age at death:

[ ]

What was the cause of death?

- Unknown
- Heart disease
- Cancer
- Cerebrovascular disease (stroke)
- Chronic lower respiratory disease
- Accident (unintentional injury)
- Diabetes
- Alzheimer disease
- Influenza and pneumonia
- Kidney disease
- Septiciemia
- Intentional self-harm (suicide)
- Chronic liver disease and cirrhosis
- Essential hypertension and hypertensive renal disease
- Parkinson disease
- Homicide
- Natural causes
- Other ____________

Was an autopsy performed?

- Yes
- No
- Unsure

If so, did the autopsy indicate Parkinson's disease?

- Yes
- No
- Unsure

Has he/she been diagnosed with Parkinson's disease by a physician?

- Yes
- No
- ____________ Unsure

If yes, was the physician a neurologist?

- Yes
- No
- Unsure

How old was he/she when the diagnosis was made?

Does he/she take Sinemet (Levodopa/Carbidopa)?

- Yes
- No
- Unsure

Does he/she exhibit any of the following symptoms? Please check all that apply.

- Tremor at rest or when sitting quiety
- Shuffling gait and/or steps that are short and tiny
- Stooped posture
- Arms that do not swing when he/she walks
- Muscular rigidity or slow/stiff movements

If he/she has any of the above symptoms, at what age did they begin?

If this person has any of the symptoms listed below, which was the first to develop? (Please select all that apply.)

- rest tremor
- action tremor
- stooped posture
- micrographia

- ☐ slowness
- ☐ stiffness
- ☐ balance problem
- ☐ shuffling gait
- ☐ decreased arm swing
- ☐ decreased facial expression
- ☐ decreased volume of speech
- ☐ dystonia
- ☐ freezing
- ☐ memory loss
- ☐ numbness
- ☐ hallucinations
- ☐ depression
- ☐ leg dragging
- ☐ other ______

Thank you. You will now be asked the same series of questions about your other siblings. If you have already completed these questions for all of your siblings, you will be asked to complete this information about your children.

Children

How many children do you have?

______

PD History: Children

Please answer the following questions for each of your children. It may be helpful to proceed from oldest to youngest, in order to keep track of which information has already been entered.

Is this person male or female?

- ○ Male
- ○ Female

Year of Birth

Does or did this person have the mutation in the Fabry gene or have symptoms of Fabry?

- Yes
- No
- I don't know

Is this person living?

- Yes
- No

Please indicate age at death:

What was the cause of death?

- Unknown
- Heart disease
- Cancer
- Cerebrovascular disease (stroke)
- Chronic lower respiratory disease
- Accident (unintentional injury)
- Diabetes
- Alzheimer disease
- Influenza and pneumonia
- Kidney disease
- Septiciemia
- Intentional self-harm (suicide)
- Chronic liver disease and cirrhosis
- Essential hypertension and hypertensive renal disease
- Parkinson disease
- Homicide
- Natural causes
- Other

Was there an autopsy performed?

- Yes
- No
- Unsure

If so, did the autopsy indicate Parkinson's disease?

- Yes
- No
- Unsure

Has he/she been diagnosed with Parkinson's disease by a physician?

- Yes
- No
- ______ Unsure

If yes, was the physician a neurologist?

- Yes
- No
- Unsure

How old was he/she when the diagnosis was made?

______

Does he/she take Sinemet (Levodopa/Carbidopa)?

- Yes
- No
- Unsure

Does he/she exhibit any of the following symptoms? Please check all that apply.

- ☐ Tremor at rest or when sitting quiety
- ☐ Shuffling gait and/or steps that are short and tiny
- ☐ Stooped posture
- ☐ Arms that do not swing when he/she walks
- ☐ Muscular rigidity or slow/stiff movements

If he/she has any of the above symptoms, at what age did they begin?

____

If this person has any of the symptoms listed below, which was the first to develop? (Please select all that apply.)

- ○ rest tremor
- ○ action tremor
- ○ slowness
- ○ stiffness
- ○ balance problem
- ○ shuffling gait
- ○ decreased arm swing
- ○ decreased facial expression
- ○ decreased volume of speech
- ○ dystonia
- ○ stooped posture
- ○ micrographia
- ○ freezing
- ○ memory loss
- ○ numbness
- ○ hallucinations
- ○ depression
- ○ leg dragging
- ○ other ________________

Thank you. You will now be asked the same series of questions about your other children. If you have already completed these questions for all of your children, you have completed the survey.

Block 10

Powered by Qualtrics
